# Supplementary material for: Molecular Characterization of the Infectious Laryngotracheitis Virus (ILTV) Involved in Poultry Outbreaks Reveals the Virus Origin and Estimated Spreading Route
Source: Viruses. 2025 Jan 31;17(2):213. doi: 10.3390/v17020213 (PMC11860664; doi:10.3390/v17020213)
Supplement: Supplementary file 1 [file viruses-17-00213-s001.zip › viruses-3388251-supplementary.pdf]

viruses

MDPI

Supplementary Materials

Molecular characterization of infectious laryngotracheitis virus (ILTV) involved in poultry outbreaks reveals virus origin and estimated spreading route

Jorge Luis Chacón [1], Ruy D. Chacón [2], Henrique Lage Hagemann [2], Claudete S. Astolfi-Ferreira [2], Cesar Nunes [1], Luiz Sesti [1], Branko Alva [1], and Antonio J. Piantino Ferreira [2,*]

[1] CEVA Animal Health, Rua Manoel Joaquim Filho, 303, São Paulo 13148115, Brazil; jorge.chacon@ceva.com (J.L.C.); cesar.nunes@ceva.com (C.N.); luiz.sesti@ceva.com (L.S.); branko.alva@ceva.com (B.A.)

[2] Department of Pathology, School of Veterinary Medicine, University of São Paulo, Av. Prof. Orlando Marques de Paiva, 87, São Paulo 05508-900, Brazil; ruychaconv@alumni.usp.br (R.D.C.); henrique.trick@alumni.usp.br (H.L.H.); csastolfi@gmail.com (C.S.A.-F.)

* Correspondence: ajpferr@usp.br; Tel.: +55-11-3091-1352

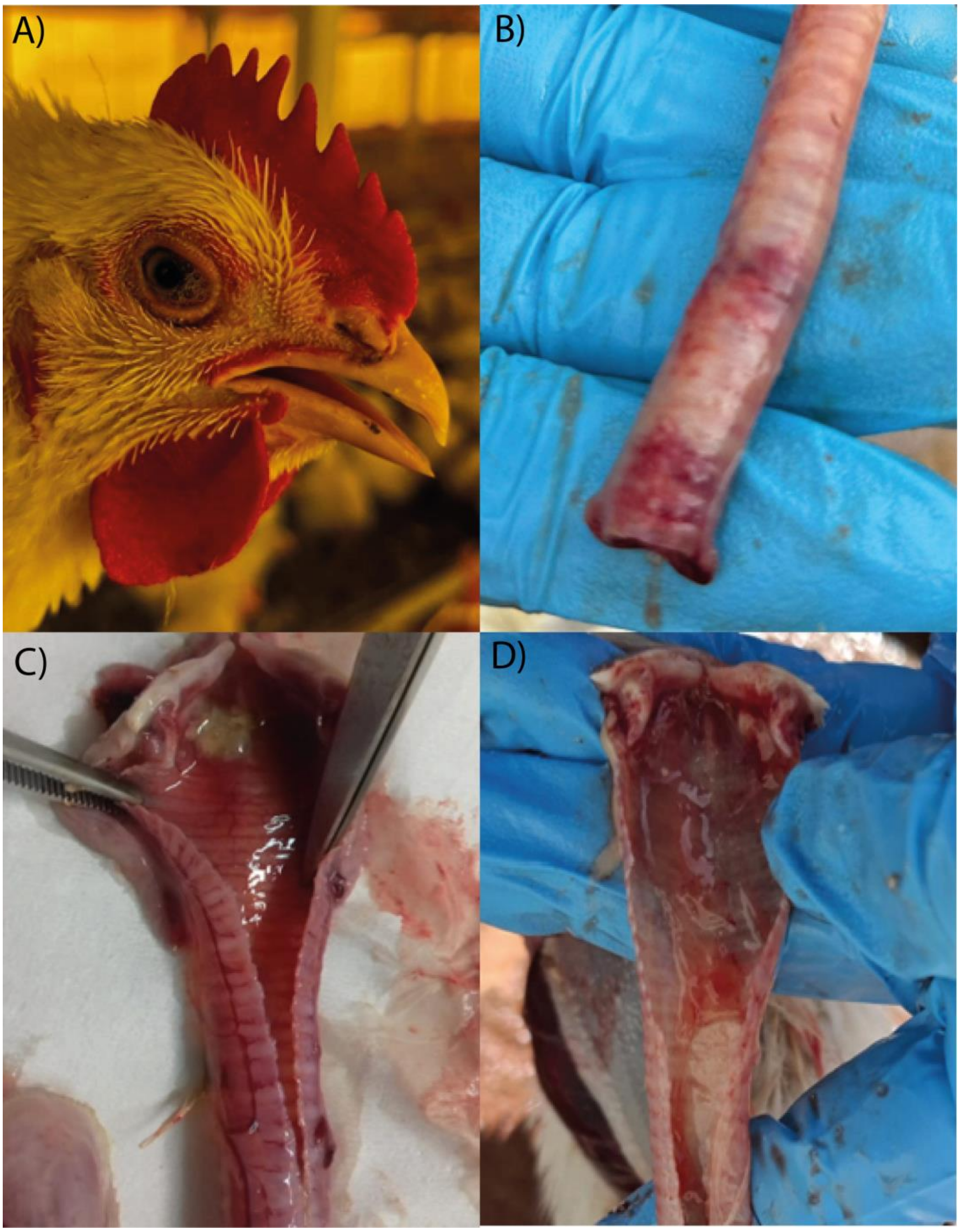

Figure S1. Gross findings in birds infected with ILTV. A) Lacrimation and swollen conjunctiva. B) Hemorrhagic content within the tracheal lumen. C) Hemorrhagic and fibrinonecrotic exudates in the larynx and trachea. D) Hemorrhagic exudates in the larynx and trachea.
